# Supplementary material for: Identification of photoperiod-induced specific miRNAs in the adrenal glands of Sunite sheep (Ovis aries)
Source: Front Vet Sci. 2022 Jul 22;9:888207. doi: 10.3389/fvets.2022.888207 (PMC9354845; doi:10.3389/fvets.2022.888207)
Supplement: Supplementary file 1 [file Data_Sheet_1.ZIP › Supplementary file/miRDeep2 file.zip!/miRDeep2 file/Know_Structure/SP42A3/oar-mir-99a.pdf]

miRBase precursor : oar-mir-99a
Total read count : 557111
oar-miR-99a read count : 556346
remaining reads : 765

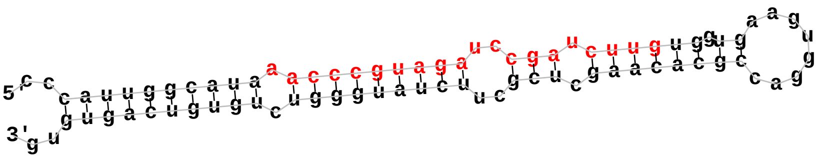

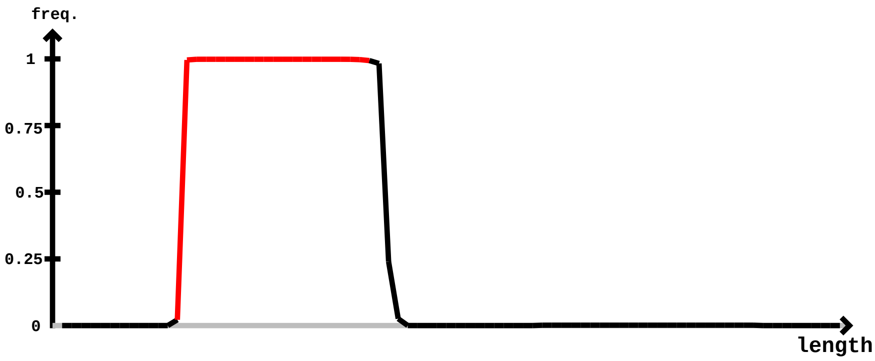


```
                oar-miR-99a
5'- cccauuggcauaaacccguagauccgaucuuguggugaaguggaccgcacaagcucgcuucuaugggucugugucagugug   -3'    exp
    ..((((((((((.(((((((((..(((.((((((.((........)))))))).)))..))))))))).))))))))))..    reads      mm         sample
    ..........uaaacccguagauccgaucuugu................................................    1          0          seq
    ...........aaacccguagauccgauc....................................................    3          0          seq
    ...........aaacccguagauccgaucu...................................................    14         0          seq
    ...........aaacccguagauccgaucuu..................................................    32         0          seq
    ...........aaacccguagauccgaucuug.................................................    101        0          seq
    ...........aaacccguagauccgaucuugu................................................    11590      0          seq
    ...........aaacccguagauccgaAcuugu................................................    4          1          seq
    ...........aaacccguagauccgaucuugug...............................................    315        0          seq
    ...........aaacccguagauccgaAcuugug...............................................    2          1          seq
    ...........aaacccguagauccgaucuugugg..............................................    24         0          seq
    ............aacccguagauccgauc....................................................    94         0          seq
    ............aacccguagauccgaAc....................................................    7          1          seq
    ............aacccguagauccgaAcu...................................................    76         1          seq
    ............aacccguagauccgaucu...................................................    600        0          seq
    ............aacccguagauccgaucuu..................................................    1816       0          seq
    ............aacccguagauccgaAcuu..................................................    172        1          seq
    ............aacccguagauccgaAcuug.................................................    949        1          seq
    ............aacccguagauccgaucuug.................................................    4946       0          seq
    ............aacccguagauccgaAcuugu................................................    21013      1          seq
    ............aacccguagauccgaucuugu................................................    380787     0          seq
    ............aacccguagauccgaAcuugug...............................................    36254      1          seq
    ............aacccguagauccgaucuugug...............................................    81761      0          seq
    ............aacccguagauccgaAcuugugg..............................................    564        1          seq
    ............aacccguagauccgaucuugugg..............................................    13627      0          seq
    ............aacccguagauccgaucuuguggu.............................................    53         0          seq
    ............aacccguagauccgaAcuuguggu.............................................    10         1          seq
    .............acccguagauccgaucu...................................................    1          0          seq
    .............acccguagauccgaAcuu..................................................    4          1          seq
    .............acccguagauccgaucuu..................................................    4          0          seq
    .............acccguagauccgaAcuug.................................................    15         1          seq
    .............acccguagauccgaAcuugu................................................    205        1          seq
    .............acccguagauccgaucuugu................................................    407        0          seq
    .............acccguagauccgaAcuugug...............................................    724        1          seq
    .............acccguagauccgaucuugug...............................................    81         0          seq
    .............acccguagauccgaAcuugugg..............................................    25         1          seq
```

oar-miR-99a

```
cccauuggcauaaacccguagauccgaucuuguggugaaguggaccgcacaagcucgcuucuaugggucugugucagugug
.............acccguagauccgaucuugugg..............................................    12     0     seq
.............acccguagauccgaucuuguggu.............................................    1      0     seq
.............acccguagauccgaAcuuguggu.............................................    1      1     seq
..............cccguagauccgaAcuugu................................................    1      1     seq
..............cccguagauccgaucuugu................................................    8      0     seq
..............cccguagauccgaucuugug...............................................    1      0     seq
...............ccguagauccgaucuugu................................................    11     0     seq
...............ccguagauccgaAcuugu................................................    1      1     seq
...............ccguagauccgaAcuugug...............................................    2      1     seq
...............ccguagauccgaucuugug...............................................    7      0     seq
................cguagauccgaucuugu................................................    8      0     seq
................cguagauccgaucuugug...............................................    5      0     seq
................cguagauccgaucuugugg..............................................    7      0     seq
.................................................caagcucgcuucuaugggucu...........    3      0     seq
.................................................caagcucgcuucuaugggucug..........    14     0     seq
.................................................caagcucgcuucuaugggucugu.........    726    0     seq
.................................................caagcucgcuucuaugggucugug........    4      0     seq
..................................................aagcucgcuucuaugggucu...........    1      0     seq
..................................................aagcucgcuucuaugggucugu.........    17     0     seq
```
